# Early-stage IgM kappa proliferative glomerulonephritis with monoclonal immunoglobulin deposition presenting with atypical renal insufficiency: a case report and literature review

**DOI:** 10.3389/fmed.2026.1918254

**Published:** 2026-08-21

**Authors:** Aoteng Cui, Jinyu Yu, Tingting Pan, Hongzhao Xu, Zhonggao Xu

**Affiliations:** 1 Department of Nephrology, The First Hospital of Jilin University, Changchun, China; 2 Department of Urology, The First Hospital of Jilin University, Changchun, China; 3 Department of Anaesthesia, The First Hospital of Jilin University, Changchun, China

**Keywords:** IgM kappa, MGRS, PGNMID, monoclonal immunoglobulin deposition, renal biopsy, C3 negative

## Abstract

**Introduction:**

Proliferative glomerulonephritis with monoclonal immunoglobulin deposits (PGNMID) is a rare subtype of monoclonal gammopathy of renal significance (MGRS). While the IgG subtype has been well characterized, the IgM-*κ* variant is extremely rare, with a low detection rate of circulating monoclonal proteins and limited real-world data on its clinical course under conservative management. This case report describes a biopsy-proven IgM-*κ* PGNMID in an elderly patient with negative hematological screening, to expand the current understanding of this rare entity.

**Patient concerns and diagnoses:**

A 70-year-old male presented with incidentally detected microscopic hematuria and elevated serum creatinine, without overt edema, gross hematuria or systemic symptoms. Renal biopsy demonstrated a membranoproliferative pattern of glomerular injury, with monoclonal IgM deposition showing kappa light chain restriction and negative C3 staining, confirming the diagnosis of PGNMID. Bone marrow biopsy revealed no clonal lymphoplasmacytic proliferation, consistent with MGRS. The patient’s serum creatinine level remained stable with a slight decrease from the baseline value, and the urinary red blood cell count returned to normal.

**Outcomes:**

After 7 months of follow-up, the patient’s serum creatinine levels remained stable with a mild decrease from baseline. This case indicates that IgM-*κ* PGNMID can present with insidious manifestations and negative conventional hematological screening. For elderly patients with unexplained hematuria and mild renal impairment, early renal biopsy is essential for early identification of rare MGRS lesions, and conservative management may be considered as an initial approach in PGNMID patients with stable mild disease.

## Introduction

Proliferative glomerulonephritis with monoclonal immunoglobulin deposition (PGNMID) is a rare but clinically significant subtype of monoclonal gammopathy of renal significance (MGRS), first formally described by Bridoux et al. (1). It accounts for approximately 0.17% of all native kidney biopsies, with a reported incidence 8-fold lower than AL amyloidosis and 2-fold lower than monoclonal immunoglobulin deposition disease (MIDD) (1). The vast majority (almost 90%) of PGNMID cases are associated with IgG deposits, predominantly the IgG3 subclass (60–68%), followed by IgG1 (24–29%) and IgG2 (3–16%). Rare variants include IgA (<5%), IgM (5–10%), and light chain-only (<1%) PGNMID (1–3). The renal prognosis of PGNMID is generally poor, with more than 50% of patients progressing to end-stage renal disease (ESRD) within 10 years of diagnosis, and early recurrence rates exceeding 80% after kidney transplantation (1, 2).

MGRS was first proposed by the International Kidney and Monoclonal Gammopathy Research Group (IKMG) in 2012 and subsequently refined in 2018. It is defined as a clonal B-cell or plasma cell disorder that produces nephrotoxic monoclonal immunoglobulins (M proteins) or their fragments, leading to kidney injury, but does not meet the diagnostic criteria for symptomatic multiple myeloma, Waldenström macroglobulinemia, or other overt hematological malignancies (4, 5). According to the 2018 IKMG consensus classification, MGRS-related renal diseases are divided into three major categories based on the site of injury and mechanism of damage: 1. Glomerular diseases: including PGNMID, C3 glomerulopathy with monoclonal immunoglobulin deposits (Ig-C3GN), MIDD, and immunotactoid glomerulopathy (ITG); 2. Tubulopathies: including light chain proximal tubulopathy (LCPT) and cast nephropathy; 3. Renal vascular diseases: including atypical hemolytic uremic syndrome (aHUS) (5). A key distinction between MGRS and hematological malignancies is that the nephrotoxicity in MGRS arises from the intrinsic pathogenic properties of the secreted M protein rather than the tumor burden itself. Most MGRS patients have less than 10% clonal plasma cells in the bone marrow, and progression to overt hematological malignancies is rare, occurring in less than 5% of cases during long-term follow-up (4–6).

IgM PGNMID is an extremely rare variant, accounting for only 5–10% of all PGNMID cases (3). Unlike IgG PGNMID, which is predominantly derived from plasma cell clones, IgM PGNMID is almost exclusively associated with CD20-positive B-cell or lymphoplasmacytic clones, making anti-CD20 monoclonal antibodies the preferred first-line treatment when clone-directed therapy is indicated (1–3). As of 2024, there have been approximately more than 20 reported cases of IgM-*κ* PGNMID in the literature, most of which present with advanced renal insufficiency, proteinuria within the nephropathy range, and require aggressive immunosuppressive treatment (3, 7, 8). We report a rare case of early-stage IgM-*κ* PGNMID in a 70-year-old male patient who presented with asymptomatic elevated serum creatinine and microscopic hematuria without detectable circulating M protein or bone marrow clonal abnormalities. The patient achieved stable renal function and complete resolution of hematuria and proteinuria with conservative treatment alone during 7 months of follow-up. This case highlights the importance of timely renal biopsy with immunoelectron microscopy for the early diagnosis of PGNMID in patients with unexplained renal insufficiency, even in the absence of detectable monoclonal gammopathy. We also review the relevant literature to discuss the clinicopathological characteristics, diagnostic challenges, and treatment strategies for this rare disease.

## Case presentation

A 70-year-old male was admitted to our hospital on May 21, 2024, for further evaluation of incidentally detected elevated serum creatinine found 4 days prior during a routine physical examination. The patient was asymptomatic with no abnormal physical signs on presentation, and had no prior history of hypertension. Blood pressure measured on admission was 169/83 mmHg. Laboratory findings on admission were as follows (Supplementary Table 1). Renal biochemical tests showed a urea level of 7.86 mmol/L, serum creatinine of 134.7 μmol/L, and uric acid of 577 μmol/L. Hemoglobin was 135 g/L, and serum albumin was 38.6 g/L. Urinalysis revealed 2 + proteinuria, 3 + hematuria, and 229.10 erythrocytes per microliter; the 24-h urinary protein excretion was 0.122 g. The 2 + proteinuria on dipstick test was considered to be affected by urine concentration and interference from significant microscopic hematuria. The 24-h urinary protein excretion (0.122 g) was used as the gold standard to evaluate the degree of proteinuria, indicating minimal proteinuria. Serum electrolyte levels were within normal ranges: potassium 4.64 mmol/L, sodium 138.8 mmol/L, calcium 2.38 mmol/L, and phosphate 1.02 mmol/L. Serum free light chain assay showed elevated free kappa light chain at 30.00 mg/L (reference range: 6.70–22.40 mg/L) and elevated free lambda light chain at 34.20 mg/L (reference range: 8.30–27.00 mg/L), with a kappa/lambda ratio of 0.877 (reference range: 0.26–1.65). Both free kappa and lambda light chains were mildly elevated with a normal kappa/lambda ratio, which was considered to be related to decreased renal clearance due to mild renal insufficiency, without evidence of circulating monoclonal light chain proliferation. The rheumatoid factor level was markedly elevated at 834.94 IU/mL. Serum IgG was 5.76 g/L, complement C3 was 0.96 g/L, and complement C4 was 0.43 g/L. Serum protein electrophoresis showed no monoclonal band, with decreased gamma globulin (8.40%). Both serum and urine immunofixation electrophoresis were negative for monoclonal immunoglobulin and light chains. Urine protein electrophoresis was not performed in this case. Fasting blood glucose, glycated hemoglobin, tumor markers, and anti-neutrophil cytoplasmic antibody (ANCA) levels were all within normal limits. Serological screening for hepatitis B virus (HBV), hepatitis C virus (HCV), syphilis, and human immunodeficiency virus (HIV) yielded negative results. Urinary tract ultrasonography showed no abnormalities in bilateral kidney size.

**Figure 1:**
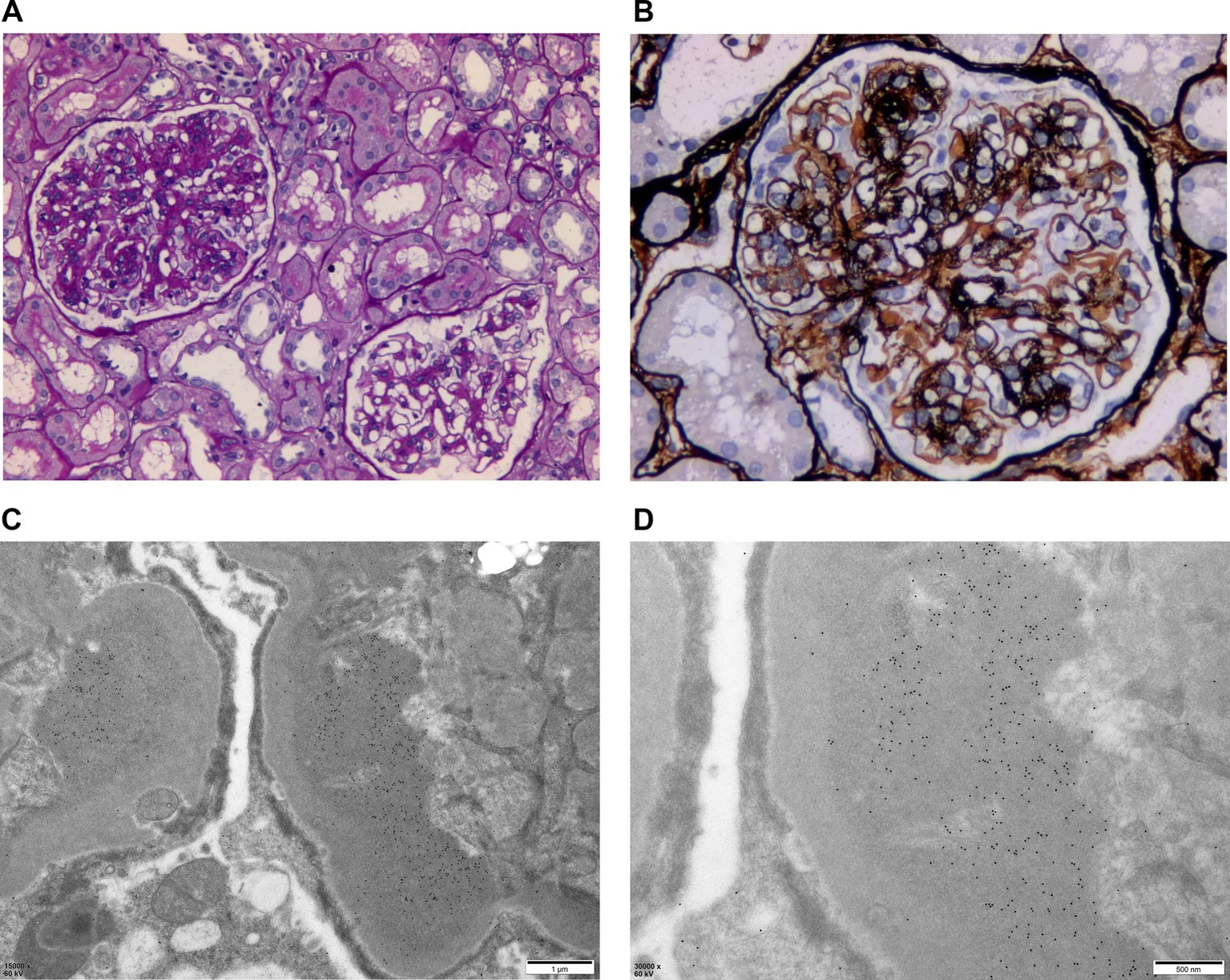
The patient’s kidney biopsy results. **(A)** Light microscopy showing diffuse mild proliferation of mesangial cells and mesangial matrix, with segmental endothelial cell swelling (Hematoxylin–eosin staining, ×200). **(B)** Periodic acid-Schiff methenamine silver (PASM) staining showing segmental duplication of glomerular basement membrane with mesangial interposition, forming the characteristic “double-track sign” (×400). **(C)** Immunoelectron microscopy showing diffuse deposition of 10 nm colloidal gold particles (anti-κ light chain antibody) in the mesangial electron-dense deposits (×15,000, bar = 1 μm). **(D)** High-magnification immunoelectron microscopy confirming that the gold particles are specifically localized within the amorphous electron-dense deposits, demonstrating κ light chain restriction (×30,000, bar = 500 nm).

Renal biopsy was performed on May 23, 2024 (Figure 1). Light microscopy revealed that the punctured renal tissue contained 16 glomeruli, with diffuse mild hyperplasia of glomerular mesangial cells and mesangial stroma; segmental hyperplasia was moderately to severely pronounced, predominantly characterized by increased mesangial stroma; segmental swelling and hyperplasia of endothelial cells; increased mesangial stroma segmentally inserted into capillary walls, presenting as membranoproliferative lesions; segmental mild thickening of the glomerular basement membrane; no definitive hemosiderin deposition or significant inflammatory cell infiltration within the glomeruli; vacuolar degeneration of renal tubular epithelial cells; occasional tubular casts; no significant tubular atrophy; sporadic focal infiltrations of lymphocytes and macrophages in the renal interstitium; no significant fibrosis in the interstitium; and mild fibrous thickening of the small arterial intima (Frozen section examination revealed 9 glomeruli without sclerosis.) Congo red special staining was negative.

Immunofluorescence on 9 glomeruli was scored on a 0 to 4 + semi-quantitative scale, showing moderate deposition of IgM (2+) and kappa (2+) along the mesangial region and some capillary walls. Staining for IgA, IgG, C3, C4, C1q, F, IgG1-IgG4, and lambda were all negative, indicating monotypic kappa light chain deposition. Electron microscopy revealed mesangial proliferative changes with amorphous electron-dense deposits within the mesangial matrix and along glomerular capillary walls, which is consistent with the pathological features of PGNMID. Combined with the immunofluorescence findings, the diagnosis favored proliferative glomerulonephritis with monoclonal immunoglobulin deposition (PGNMID). Immunoelectron microscopy confirmed kappa restriction (*κ*++/*λ*-), supporting PGNMID of kappa type.

After discharge, the patient underwent a bone marrow biopsy in the Department of Hematology at Peking Union Medical College Hospital. The posterior iliac crest bone marrow biopsy revealed small amounts of bone tissue and bone marrow tissue. The proportion of hematopoietic tissue to fat in the bone marrow sample was approximately normal. The granuloid-to-erythroid ratio was generally within the normal range. Megakaryocytes were present. Immunohistochemistry results: CD3 (scattered positive), CD20 (occasional single positive cells), CD38 (scattered positive), CD79a (scattered positive), CD138 (occasional single positive cells), Ki-67 (index 90%), Mum-1 (scattered positive), PAX-5 (scattered positive), CD15 (predominantly positive), CD61 (scattered positive), CD56 (negative), CD71 (weakly positive in a small subset). *In situ* hybridization: kappa ISH (negative), lambda ISH (negative); positive control (+), negative control (−). The proportion of plasma cells in the bone marrow tissue was less than 5%, with no evidence of clonal lymphoplasmacytic proliferation.

**Figure 2:**
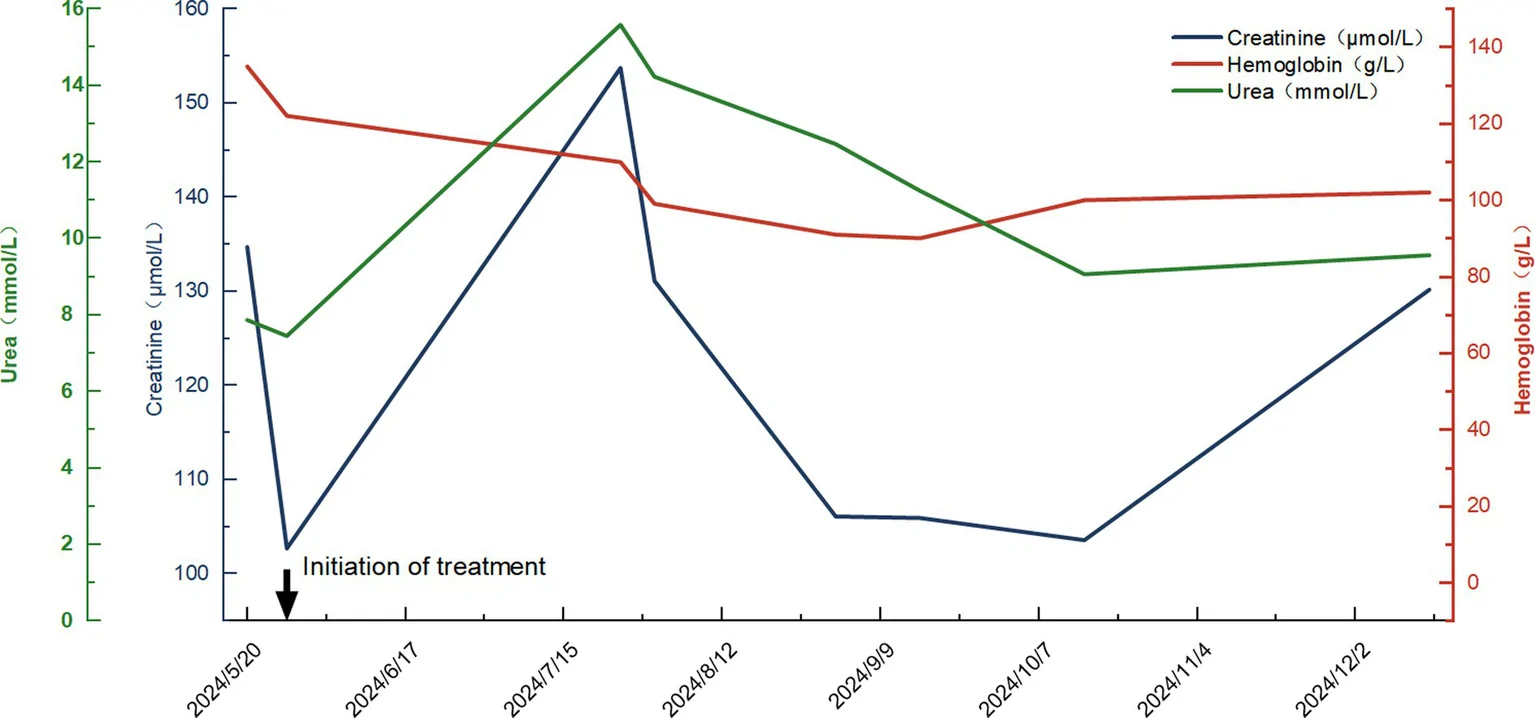
Clinical course of the patient after admission. The x-axis represents follow-up time from May to December 2024. The left dual y-axes denote serum creatinine (μmol/L, blue line) and serum urea (mmol/L, green line), respectively. The right y-axis denotes hemoglobin concentration (g/L, red line). The black arrow indicates the time point of treatment initiation (May, 2024).

The patient received renal supportive therapy including uric acid lowering, blood pressure control and renal protection after discharge. During follow-up, the patient’s serum creatinine level remained stable with a slight decrease from the baseline value, and the urinary red blood cell count returned to normal (Figure 2).

## Discussion

PGNMID is one of the most diagnostically challenging subtypes of MGRS, presenting with nonspecific renal manifestations such as hematuria, proteinuria, hypertension, and renal insufficiency. Unlike other forms of MGRS, approximately 70% of patients with PGNMID have negative serum protein electrophoresis (SPEP) and serum immunofixation electrophoresis (SIFE) for M proteins; this seronegative profile can readily obscure the presence of severe kidney injury (5). This phenomenon may be attributable to the predominant deposition of pathogenic monoclonal immunoglobulin in the kidney and its low circulating concentration below the detection threshold. A negative serum M protein result therefore cannot be used to exclude PGNMID. The diagnosis of PGNMID rests entirely on kidney biopsy, with typical pathological features including a membranoproliferative glomerulonephritis pattern, monoclonal immunoglobulin deposits, and granular electron-dense deposits, and requires the exclusion of cryoglobulinemia (1). In the present case, the serum free light chain ratio was normal and both SPEP and SIFE were negative; however, kidney biopsy revealed deposits of IgM and *κ* light chains along the glomerular mesangium and capillary walls, confirming the diagnosis of IgMκ PGNMID.

IgMκ PGNMID is exceedingly rare, with only approximately 20 cases reported to date (Table 1), seven of which had concurrent deposition of IgG heavy chains or *λ* light chains (3, 7–12). A systematic review of these cases revealed that the patients with this disease were mainly middle-aged and elderly men. The most common pathological type was membranoproliferative glomerulonephritis, with complement C3 positivity being the majority, and the positive rate of serum M protein was approximately 60%. Compared with the reported cases, the degree of proteinuria in this patient was milder (24-h urine protein 0.122 g), and it was a rare case of C3 negative PGNMID. After conservative treatment, the renal function improved in a short period of time. The above characteristics not only reveal the significant clinical heterogeneity of IgMκ PGNMID, but also suggest that patients with early-stage and mild disease may not need to immediately initiate immunosuppressive therapy.

**Table 1:** Summary of previous reports of IgM Kappa PGNMID and their clinical characteristics.

Reference	Age/Sex	Pathologic pattern	Glomerular deposits (IF)	C3	Treatment	Renal outcome/follow-up
Lima et al. (9)	76/M	Mesangial proliferative, 40% crescents	Serum IgMκ (kidney IF not stained for κ)	Negative	Methylprednisolone+chlorambucil	Stable sCr 2 mg/dL, UP <1 g/24 h; 12 months
van Kruijsdijk et al. (10) Case 1	53/F	Mesangial + endocapillary proliferation	IgM (1–2+), κ (IHC κ > *λ*)	C3 (2+)	None (continued AZA + pred)	Stable sCr, no hematuria/proteinuria; >3 years
van Kruijsdijk et al. (10) Case 2	34/F	Mild mesangial proliferation	IgM (3+), κ (2+), λ (−)	C3 (1–2+)	B + D (BRD stopped due to AE)	Resolved hematuria/proteinuria, stable sCr; >3 years
Oe et al. (18)	48/M	MPGN	IgM (3+), κ (2+), λ (−)	C3 (2+)	R-FC × 4period	Proteinuria ↓, sCr 1.5 mg/dL
Ramos et al. (11)	48/M	MPGN, 40% crescents	IgMκ	C3 (+)	Pred+Chlorambucil×1 year	Persistent proteinuria, ESRD at 2 years
Yamaguchi et al. (8)	53/M	MPGN-like	IgMκ λ (±)	C3 (weak +)	ARB alone	eGFR 65.7 → 64.2, UP 3.5–3.8 g/d; 3 years
Chen et al. (7)	56/M	MPGN	IgM (++), κ (++), λ (±)	C3 (2 + −3+)	Pred acetate	eGFR 51.65, UP 1.29 g/d
Bu et al. (3) #5	72/M	Early MPGN	κ-restricted (MALDI-TOF IgMκ)	Not reported	Chlo+Obi→R + Cy + Pred→Ibr → R	ESKD; 78 months
Bu et al. (3) #9	82/M	EPGN	κ-restricted (free κ on SPEP)	Not reported	Pred+R	Persistent renal impairment (sCr 4.2, UP 1.3); 68 months
Bu et al. (3) #10	72/M	MesPGN	IgMκ	Not reported	Pred	Persistent renal impairment (sCr 3.0, UP 0.1); 30 months
Bu et al. (3) #22	71/F	MPGN	IgMκ	Not reported	R	Complete recovery (sCr 1.15, UP 0.02); 17 months
Gumber et al. (12)	65/M	MPGN	IgMκ	Not reported	R-Cy-Pred	PR
Gumber et al. (12)	78/M	MPGN	IgMκ	Not reported	None	Not reported

BM, bone marrow; IF, immunofluorescence; IHC, immunohistochemistry; MPGN, membranoproliferative glomerulonephritis; sCr, serum creatinine; UP, urine protein; AZA, azathioprine; pred, prednisone; B, bortezomib; D, dexamethasone; R, rituximab; BRD, bortezomib + rituximab + dexamethasone; AE, adverse events; PN, peripheral neuropathy; R-FC, rituximab + fludarabine + cyclophosphamide; ESRD, end-stage renal disease; ARB, angiotensin receptor blocker; eGFR, estimated glomerular filtration rate; IFE, immunofixation electrophoresis; Chlo, chlorambucil; Obi, obinutuzumab; Cy, cyclophosphamide; Ibr, ibrutinib; R-Cy-Pred, rituximab + cyclophosphamide + prednisone; PR, partial remission.

The detection rate of clonal plasma cells on bone marrow biopsy in patients with PGNMID is similarly low, ranging from only 20 to 30% (2). The absence of detectable clonal cells in the bone marrow at diagnosis does not completely exclude the existence of a potential underlying B-cell or plasma cell clone. A minority of PGNMID patients may develop overt plasma cell dyscrasia or B-cell lymphoma during long-term follow-up. Therefore, regular hematological monitoring is necessary even for patients with initially negative bone marrow results. Nevertheless, the deposition of monoclonal immunoglobulin in the kidney arises from the clonal proliferation and secretion of B lymphocytes or plasma cells, suggesting a potential underlying hematologic disorder and accounting for the high recurrence rate of PGNMID after kidney transplantation; early immunosuppressive therapy may reduce the risk of recurrence and prolong survival (13, 14).

For patients with PGNMID who have a negative bone marrow biopsy, current treatment strategies fall into three principal categories: conservative management, clone-directed therapy, and non-clone-directed therapy. Conservative management is generally reserved for patients with 24-h urine protein <1 g, no evidence of disease progression, and CKD stage 1 or 2, in whom spontaneous renal remission may occasionally occur (4, 5, 13). For this patient, the decision for conservative management was made based on four factors. First, the patient had early-stage mild disease with minimal proteinuria and no active pathological lesions on biopsy. Second, no clonal proliferation was detected in the bone marrow, and there was no clear target for clone-directed therapy. Third, this approach aligned with current consensus recommendations for managing mild stable MGRS/PGNMID. Finally, a risk–benefit assessment for this elderly patient favored close monitoring over upfront immunosuppression, a choice made after obtaining informed consent. Non-clone-directed therapy (e.g., glucocorticoids, mycophenolate mofetil, cyclophosphamide) is indicated for patients without clonal evidence but with active or progressive disease; however, its renal response rate is lower than that of clone-directed therapy (4). Clone-directed therapy is tailored to the cellular target: rituximab is preferred for B-cell clones, whereas bortezomib or daratumumab can be used for plasma cell clones; this approach is especially appropriate for patients with definitive clonal evidence or progressive renal decline. Given that IgM-type PGNMID is strongly associated with underlying CD20-positive B-cell lymphoproliferative disorders, systemic screening including peripheral blood flow cytometry and whole-body imaging should be recommended for all patients with IgM PGNMID, even in the absence of obvious clinical manifestations (12).

However, existing data suggest that conservative management may have a very limited role. Bu et al. reported 23 cases of IgM PGNMID, among whom none of the four patients receiving supportive care alone achieved renal remission, whereas some of those treated with rituximab showed partial responses, including one patient who attained a complete remission (3). These findings indicate that conservative management may only be suitable as an initial strategy for selected patients with very early and mild disease, underscoring the critical importance of long-term follow-up and close monitoring of renal function and proteinuria. Should proteinuria increase to >1 g/d or renal function decline progressively during follow-up, clone-directed therapy should be initiated promptly, with rituximab as the preferred agent for IgM PGNMID (15). Short-term stable renal function in this case does not guarantee long-term prognosis, which further emphasizes the necessity of close long-term monitoring for all PGNMID patients.

The conventional view holds that the core pathogenesis of PGNMID involves complement activation triggered by the deposition of monoclonal immunoglobulin in the glomeruli, leading to inflammatory cell infiltration, mesangial cell proliferation, and basement membrane injury (1). In IgG3 PGNMID, which accounts for the vast majority of cases, C1q deposition is highly prevalent; in contrast, among IgM cases, the C1q deposition rate is only 17%, whereas C3 deposition reaches 96%, suggesting that IgM PGNMID may activate complement predominantly via the alternative or lectin pathway (3). However, both the present case and the case reported by Lima et al. (9) exhibited completely negative C3 staining, strongly pointing to a complement-independent pathogenic mechanism. IgM immunoglobulins are produced by a single B-cell/plasma-cell clone, and their monoclonal IgM molecules possess a uniform, fixed structure (16). This structural uniformity may confer distinct molecular properties. Drawing on structural biology studies of IgM (16), we hypothesize that the following complement-independent mechanisms may operate in C3 negative IgMκ PGNMID: the deposited monoclonal IgM may bind to specific receptors on mesangial cells or glomerular endothelial cells, directly activating intracellular signaling pathways that induce cell proliferation and extracellular matrix secretion; additionally, the possibility cannot be entirely excluded that complement activation occurs below the detection threshold of immunofluorescence and contributes to local inflammation (17). These observations suggest that PGNMID may be divided into two subtypes: “complement-dependent” and “complement-independent.” In patients with the complement-independent subtype, the use of complement inhibitors lacks a theoretical rationale, and treatment should focus on eliminating the pathogenic immunoglobulin or its producing cells (e.g., rituximab or bortezomib) rather than the indiscriminate use of complement blockers. Naturally, these mechanistic hypotheses await further validation through *in vitro* experiments, animal models, and additional clinical cases.

Our study has several limitations. First, the follow-up duration in our center was only 7 months, and the patient was subsequently lost to follow-up, so the long-term renal and hematological outcomes remain unclear. Second, whole-body PET-CT and peripheral blood flow cytometry were not performed at initial presentation, which may have missed potential underlying B-cell lymphoproliferative disorders. Third, urine protein electrophoresis was not performed, which may have limited the detection of low-level urinary monoclonal proteins. Despite these limitations, the diagnostic value of this rare case remains clinically significant.

A review of the existing literature confirms that the overall incidence of PGNMID is low, and the IgMκ subtype is even rarer. During the diagnostic workup of this case, we systematically excluded conditions with similar clinical or pathological presentations, including cryoglobulinemia and light chain deposition disease, before establishing the definitive diagnosis of IgMκ PGNMID. Given that the patient was in an early stage of the disease with only mild renal injury, and following thorough discussion with the patient, we opted to prioritize conservative management in the hope of achieving spontaneous recovery of renal function. This case clearly illustrates that for patients presenting to the nephrology department with atypical renal manifestations, timely kidney biopsy plays a critical role in establishing the diagnosis and guiding subsequent management.

## Data Availability

The original contributions presented in the study are included in the article/Supplementary material, further inquiries can be directed to the corresponding authors.
