# Supplementary material for: Early-stage IgM kappa proliferative glomerulonephritis with monoclonal immunoglobulin deposition presenting with atypical renal insufficiency: a case report and literature review
Source: Front Med (Lausanne). 2026 Aug 21;13:1918254. doi: 10.3389/fmed.2026.1918254 (PMC13541449; doi:10.3389/fmed.2026.1918254)
Supplement: Supplementary file 1 [file Table_1.docx]

Supplementary Table 1: Initial laboratory investigation upon admission

| Items | Value | Normal range | Units |
| --- | --- | --- | --- |
| Blood biochemical indicators | | | |
| Serum creatinine | 134.7 | 58-110 | μmol/L |
| Urea | 7.86 | 3.6-9.5 | mmol/L |
| Uric acid | 577 | 208-428 | μmol/L |
| Serum albumin | 38.6 | 40.0-55.0 | g/L |
| AST | 17.8 | 15.0-40.0 | U/L |
| ALT | 7.6 | 9.0-50.0 | U/L |
| Blood tests | | | |
| Leukocyte | 4.63 | 3.5-9.5 | ×10^9/L |
| Hemoglobin | 135 | 115-150 | g/L |
| Platelet | 172 | 125-350 | ×10^9/L |
| Electrolyte |  |  |  |
| Potassium | 4.14 | 3.5–5.3 | mmol/L |
| Sodium | 140.5 | 137–147 | mmol/L |
| Chlorine | 103.5 | 99–110 | mmol/L |
| Phosphorus | 1.02 | 0.85–1.51 | mmol/L |
| Calcium | 2.38 | 2.2–2.65 | mmol/L |
| Cardiac function | | | |
| NT-proBNP | 127 | 0-900 | pg/ml |
| Myoglobin | 48 | 0-121 | ng/mL |
| CKMB | 0.39 | 0-3.38 | ng/ml |
| Troponin I | <0.012 | 0-0.034 | ng/mL |
| Electrophoresis | | | |
| Albumin | 64.94 | 55.19-69.98 |  |
| α1 globulin | 4.47 | 2.68-5.03 | % |
| α2 globulin | 7.95 | 4.93-10.19 | % |
| β globulin | 14.24 | 6.70-15.57 | % |
| γ globulin | 8.40 | 9.81-19.81 | % |
| Alb:Globulin | 1.85 | 1.23-2.33 | % |
| Immune-related indicators | | | |
| IgA | 3.54 | 1.0-4.2 | g/L |
| IgG | 5.76 | 8.6–17.4 | g/L |
| IgM | 1.30 | 0.3–2.2 | g/L |
| C3 | 0.96 | 0.7–1.4 | g/L |
| C4 | 0.43 | 0.1–0.4 | g/L |
| CRP | 0.91 | 0-1.0 | mg/L |
| RF | 834.94 | 0-14.00 | IU/mL |
| ASO | 35.20 | 0-200 | IU/mL |
| ESR | 23 | 0-20 | mm/h |
| Coagulative function | | | |
| PT | 10.6 | 9.0-13.0 | s |
| APTT | 26.7 | 21-33 | s |
| D-dimer | 0.16 | 0.00-0.50 | mg/L FEU |
